# Occurrence and Characterization of Wheat Streak Mosaic Virus Found in Mono- and Mixed Infection with High Plains Wheat Mosaic Virus in Winter Wheat in Ukraine

**DOI:** 10.3390/v14061220

**Published:** 2022-06-03

**Authors:** Illia Pozhylov, Halyna Snihur, Tetiana Shevchenko, Irena Budzanivska, Wenwen Liu, Xifeng Wang, Oleksiy Shevchenko

**Affiliations:** 1 Virology Department, ESC “Institute of Biology and Medicine”, Taras Shevchenko National University of Kyiv, 01601 Kyiv, Ukraine; illiapozhylov@gmail.com (I.P.); galya_snigur@yahoo.com (H.S.); tyvonchuk@ukr.net (T.S.); birishechka68@gmail.com (I.B.); 2 Laboratory of Plant Viruses, D.K. Zabolotny Institute of Microbiology and Virology of the National Academy of Sciences of Ukraine, 03143 Kyiv, Ukraine; 3 State Key Laboratory for Biology of Plant Diseases and Insect Pests, Institute of Plant Protection, Chinese Academy of Agricultural Sciences, Beijing 100193, China; liuwenwen01@caas.cn

**Keywords:** wheat streak mosaic virus, High Plains wheat mosaic virus, identification, phylogenetic analysis, *Triticum aestivum*, potyvirus, emaravirus

## Abstract

Although wheat streak mosaic virus (WSMV) is a well-known pathogen inducing significant crop losses and endangering wheat production worldwide, the recent discovery of High Plains wheat mosaic virus (HPWMoV) in Ukraine raises questions on the co-existence of these two viruses having a similar host range and the same mite vector. Here we report on the screening of winter wheat industrial plantings in several important regions of Ukraine for WSMV and HPWMoV. WSMV was identified in an extremely high number of symptomatic plants (>85%) as compared to HPWMoV detected in 40% of wheat samples. Importantly, the preferred mode of HPWMoV circulation in Ukraine was mixed infection with WSMV (>30%) as opposed to WSMV, which was typically found in monoinfection (60%). Screening wheat varieties for possible virus resistance indicated that all but one were susceptible to WSMV, whereas over 50% of the same varieties were not naturally infected with HPWMoV. Overall, phylogenetic analysis of the collected WSMV and HPWMoV isolates indicated their high identity and similarity to other known isolates of the respective viruses. Here we first characterize WSMV isolates found in winter wheat plants in mono- or mixed infection with HPWMoV, which was recently reported as a typical wheat pathogen in Ukraine.

## 1. Introduction

Thanks to its high yields on organic-rich chernozem soils, *Triticum aestivum* L., and especially winter wheat, remains the second-most common grain cereal crop cultivated both in Ukraine and worldwide for centuries and is outcompeted only by corn.

Depending on the year, winter wheat crops in Ukraine reach 6.4 to 7.3 million hectares and are of strategic importance for ensuring food safety and export trade. Viral diseases are major factors causing a significant reduction in wheat yields. Available evidence indicates that, nowadays, wheat streak mosaic virus (WSMV) is one of the most common and harmful wheat pathogens common in Europe, the United States, Canada, Australia, the Middle East, North Africa, Asia, and other wheat-growing regions of the world [1,2,3,4].

WSMV is a type species of *Tritimovirus* genus of *Potyviridae* family, and has filamentous particles of ~700 nm in length and ~13 nm in diameter [5]. WSMV genome is composed of a single-stranded positive RNA molecule of 9339–9384 nucleotides (nt) encoding a single polyprotein, which is subsequently processed by virus-encoded proteases into functional proteins [6,7].

WSMV can naturally infect many species of plants of the *Poaceae* family, which are routinely cultivated in Ukraine, including all wheat cultivars/varieties (*T. aestivum* L.). Most WSMV isolates can also infect oat (*Avena sativa* L.), barley (*Hordeum vulgare* L.), rye (*Secale cereale* L.), some cultivars/varieties of corn (*Zea mays* L.), millet (*Panicum miliaceum* L.), and various grasses both among cultivated plants and wild flora acting as reservoir plants and ensuring overwintering of the virus [4,8,9,10].

On grain cereals, WSMV typically causes symptoms of streak mosaic, but may also induce necrosis of shoots and overall plant stunting. Visual symptoms of the disease depend on the virus strain, species, and cultivar/variety of the infected plant, the period of infection, temperature, and other environmental conditions [11,12]. This virus is known to damage the reproductive organs of the plants leading to the deterioration of sowing qualities of seeds and reduced germination energy and germination efficiency (almost by 50%). The general harmfulness of wheat streak disease depends on the weather conditions, the cultivar of a particular crop, the sowing period, the population density of the mite vector, and agronomic techniques [12,13,14,15,16,17,18]. WSMV is considered to be a major factor limiting wheat cultivation in Texas [19] and causes significant damage to spring and winter wheat. In combination with triticum mosaic virus (TriMV) and High Plains wheat mosaic virus (HPWMoV, also known as High Plains virus (HPV), wheat mosaic virus (WMoV), or maize red stripe virus (MRSV/MRStV)), WSMV causes annual crop losses of approximately 5%, while in epidemic years, such co-infection may cause yield losses of up to 100% in the Great Plains (USA) [20,21,22].

In this complex, TriMV is another representative of the *Poacevirus* genus of the *Potyviridae* family with filamentous particles of 680–750 nm in length and 12–15 nm in diameter. In turn, HPWMoV belongs to the *Emaravirus* genus of the *Fimoviridae* family and has double-membrane spherical particles 80–200 nm in diameter [23].

WSMV, HPWMoV, and TriMV are all efficiently transmitted from infected to healthy plants by the wheat curl mite (*Aceria tosichella* Keifer, also known as *Aceria tritici*, Acarina: *Eriophyidae*), the single known vector for these viruses [17,24]. WSMV is the only wheat virus known to be mechanically transmitted. In addition, many researchers demonstrated that WSMV can also be transmitted by seed. Seed transmission was shown to depend on wheat genotype and varied from 0.03–0.06% [25] to 0.22% [26,27] allowing rather high numbers of potentially virus-contaminated seeds sown. HPWMoV was also shown to be transmitted by sweet corn seed, however with a low frequency [28,29].

Coat protein gene-based phylogenetic analysis of WSMV isolates found in Australia suggested that the virus may have invaded Australia through the virus-contaminated selection wheat seed imported from the United States [2]. Consequently, WSMV was found in sweet corn seed imported from Japan and wheat seed from the United States [30]. No correlation was shown between the occurrence of WSMV in the seed material (i.e., probability of seed transmission of the virus) and the size of the seeds [27]. Therefore, the separation of poor (thin) seeds cannot be used for the elimination of virus-contaminated seeds in seed production.

Four phylogenetic groups of WSMV are currently recognized: A (Mexico), B (Europe, Russia, and Asia), C (Iran and Middle Asia), and D (the USA, Argentina, Brazil, Australia, Turkey, and Canada) [31].

The resistance genes *Wsm1*, *Wsm2,* and *Wsm3* have been identified insofar. Recently, the most promising candidate gene, *Wsm2*, was successfully introduced into the germline of several wheat varieties [32]. However, the data on the importance of resistance genes for practical WSMV control in the field is lacking and requires more research, which should also be focused on the field resistance of available wheat cultivars.

Outbreaks of the disease caused by WSMV in wheat crops have occasionally occurred in Ukraine [13,14,15,16,32]. Historically, WSMV was mainly spread in the eastern part of Ukraine, but available data suggest that WSMV may now be considered the most prevalent cereal virus in Ukraine, having ‘replaced’ barley yellow dwarf virus, which was dominant back in the 1990s [33,34]. In addition, recently, we have also confirmed the circulation of HPWMoV in cereal crops in several eastern regions of Ukraine, which was the first report of this pathogen detected in Europe [35]. As WSMV can possibly co-infect wheat plants in combination with HPWMoV and other virus(es) inducing similar symptoms, here we identified and evaluated the spread of WSMV in agriecosystems of Ukraine to establish patterns of its circulation in winter wheat (mono- and/or mixed infection), the geographical spread of the pathogen(s), wheat cultivar susceptibility, as well as phylogenetic properties of identified virus isolates found in mono- or mixed infection.

## 2. Materials and Methods

### 2.1. Sample Collection

Winter wheat plant samples with visual symptoms typical of WSMV, BSMV, HPWMoV, and TriMV (i.e., streak/stripe mosaic, plant stunting) were collected in May–June of 2017–2019. Sampling locations included crop-producing areas in Kyiv, Cherkassy, Vinnytsia, Dnipropetrovsk, Donetsk, Luhansk, Zaporizhia, Kharkiv, and Odessa regions.

### 2.2. Enzyme-Linked Immunosobent Assay (ELISA)

Samples collected from symptomatic plants were tested for WSMV and HPWMoV infection by a double-antibody sandwich enzyme-linked immunosorbent assay (DAS-ELISA), as described by Clark and Adams [36], using specific diagnostic kits from Loewe Biochemica GmbH (Sauerlach, Germany) and Agdia (Elkhart, IN, USA), respectively, and following the manufacturers’ recommendations. Because barley stripe mosaic virus (BSMV) in wheat induces symptoms similar to those of WSMV, the samples were also tested for BSMV presence using a kit from Loewe Biochemica GmbH (Sauerlach, Germany) to exclude mixed infection. The analysis was performed on three replicates. Briefly, ~1–2 g of leaf tissue was ground to a powder with a mortar and pestle in phosphate-buffered saline, pH 7.4 (1:10, *m*/*v*), with the addition of 2% polyvinyl pyrrolidon, 0.05% Tween 20, and 0.2% dried milk. Sap from healthy plants was used as negative controls. Positive controls were commercial protein preparations from respective manufacturers. MICROCOLON™ microtitre plates (Greiner Bio-One GmbH, Kremsmünster, Austria) were coated with virus-specific polyclonal antibodies in carbonate buffer, pH 9.6. Leaf extracts were then added to the plates in wells and incubated overnight at 4 °C. Virus presence in the samples was detected in 200 μL homogenate by virus-specific antibodies conjugated to alkaline phosphatase using a p-nitrophenyl phosphate substrate (Loewe Biochemica GmbH, Sauerlach, Germany). Absorbance values at 405 nm were measured using a Termo Labsystems Opsis MR microtiter plate reader (Thermo Fisher Scientific, Waltham, MA, USA) with Dynex Revelation Quicklink software v.4.2. Absorbance values measured 60 min after adding the substrate, greater than three times those of the negative controls, were considered positive.

### 2.3. Transmission Electron Microscopy (TEM)

Transmission electron microscopy for direct virus indication and studying virus morphology was conducted employing standard techniques of negative contrasting for clarified virus preparations. Briefly, copper grids (Sigma, St. Louis, MO, USA) were coated with chloroform-dissolved 0.2% polyvinyl formaldehyde (Serva, Heidelberg, Germany) and dried overnight on filter paper at room temperature. Then the coated grids were deposited in the clarified homogenate of symptomatic plant sap, incubated for 2–15 min at room temperature, and dried on filter paper for 1 min. The samples deposited onto grids were stained with 2% uranyl acetate (Serva, Heidelberg, Germany) for 10 min, and examined using a JEM 1400 (JEOL, Akishima, Japan) transmission electron microscope [37,38]. The samples were photographed at a magnification of 5000–35,000×.

### 2.4. RNA Extraction, Reverse Transcription-Polymerase Chain Reaction (RT-PCR), and Sequencing

Total RNA was extracted from fresh leaves of virus-infected wheat plants using a Gene JET RNA Purification kit (Thermo Fisher Scientific, Waltham, MA, USA) and following the manufacturer’s instructions.

One-step RT-PCR was performed using a Qiagen one-Step RTPCR kit + Q-solution (Qiagen, Germantown, MD, USA) according to the manufacturer’s instructions. Amplification was performed using Genetic Research Instrumentation Ltd. thermocycler (Braintree, United Kingdom). WSMV-specific oligonucleotide primers for amplifying a part of the coat protein gene [39], as well as HPWMoV-specific probes amplifying a part of the nucleoprotein gene [40] and TriMV-specific primers amplifying a PIPO region [41], were used (Table 1).

The amplification reactions were set up as follows: Initial denaturation for 3 min at 94 °C, followed by 35 cycles of denaturation at 94 °C for 30 s, annealing at 55 °C/53 °C/45 °C (WSMV/HPWMoV/TriMV) for 30 s, and extension at 72 °C for 1 min. The final extension was at 72 °C for 7 min. PCR products with GeneRuler 100 bp Plus DNA Ladder (Thermo Fisher Scientific, Waltham, MA, USA) or PCRBIO Ladder III 50 bp DNA Marker (Witec AG, Sursee, Switzerland) were separated in a 1.5% agarose gel stained with ethidium bromide, and visualized under UV light [42,43].

Fragments of the expected size were excised from the gels and cleaned using a QIAquick Gel Extraction Kit (Qiagen, Düsseldorf, Germany) following the manufacturer’s instructions. Purified amplicons were sequenced using the cycle sequencing technology on Applied Biosystems ABI 3730X1 DNA Analyzer (Applied Biosystems, Foster City, CA, USA) using the Big Dye Terminator v3.1 Cycle Sequencing kit (Thermo Fisher Scientific, Waltham, MA, USA).

### 2.5. Phylogenetic Analysis

The resulting sequences were aligned using Clustal W v.2.0 and further compared with the nucleotide sequences of respective viruses publicly available from the GenBank database (http://www.ncbi.nlm.nih.gov, accessed on 1 April 2022) using BLAST (http://blast.ncbi.nlm.nih.gov/Blast.cgi, accessed on 1 April 2022)). For phylogenetic analysis, the Neighbor-Joining (NJ) method was employed with the Jukes–Cantor model [44,45]. Branch support was evaluated by bootstrap analysis based on 1000 pseudoreplicates. The inferred trees were displayed using MEGA 7 software [46,47]. Nucleotide and amino acid similarities were estimated using the Kimura two-parameter method [48] and the Dayhoff PAM250 matrix [49], respectively.

### 2.6. Statistical Analysis

Five biological repeats were conducted per each ELISA measurement. Each biological repeat contained leaves from an individual plant. Three technical repeats were conducted for each biological repeat. When appropriate, the technical repeat data were averaged to obtain the mean value for each biological repeat. Data processing was performed using Microsoft Office Excel v.14.7.3 (Microsoft, Redmond, WA, USA). For phylogenetic analysis, in-built algorithms of MEGA 7 software were used.

## 3. Results

### 3.1. Surveys and Virus Detection by DAS-ELISA

**Figure 1:**
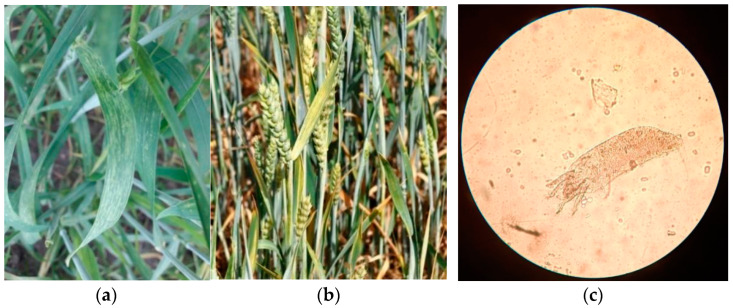
Symptoms of leaf mosaic on winter wheat plants naturally monoinfected with wheat streak mosaic virus (WSMV) only (**a**) and mixed infected with WSMV + High Plains wheat mosaic virus (HPWMoV) (**b**), and gall mite found on wheat plants (instrumental magnification ×625) (**c**).

In May-June of 2017–2019, several regions of Ukraine representing ~60% of the total area of winter wheat production were visually screened for signs of viral diseases. Wheat plants exhibiting typical virus-like symptoms, in particular leaf blade mosaic and stunting (Figure 1), were collected for subsequent analysis. Notably, *A. tosichella* (*A. tritici*) mites were often spotted on such and/or neighboring plants during sampling in most locations, suggesting a means for possible vectored virus transmission.

Judging by the symptoms it was presumed that such plants could have been infected by WSMV or BSMV in the form of either mono- or mixed infection. Selected samples were further serologically tested using DAS-ELISA.

In 2017, WSMV was found to be widespread and circulating in 6/7 screened regions of Ukraine, with an overall incidence of 71.4% in symptomatic plants (50/70). Furthermore, BSMV was not detected (Table 2).

However, in 2017, there was a remarkable portion of WSMV/BSMV-negative samples characterized by severe stripe mosaic symptoms often accompanied by notable stunting, which indicated the possible presence of (an)other virus(es) inducing a similar pathology in wheat. Hence, wheat samples collected subsequently in 2018 and 2019 were also tested for HPWMoV. Because of the absence of ELISA-based diagnostic kits for TriMV detection, this pathogen was omitted at the stage of serological testing.

**Figure 2:**
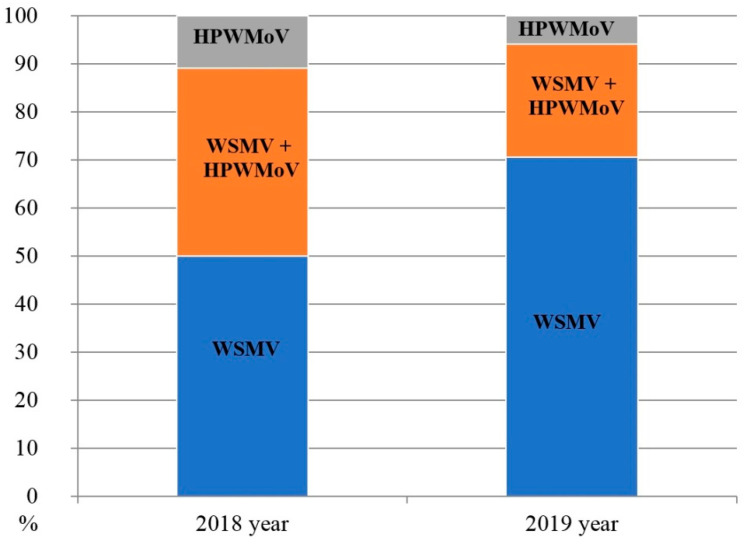
Dynamics of infection patterns induced by wheat streak mosaic virus (WSMV) and High Plains wheat mosaic virus (HPWMoV) in winter wheat in 2018 and 2019.

In 2018, serological testing of symptomatic wheat samples showed the occurrence of WSMV in every region probed (6/6) with an incidence of 88.9% in symptomatic plants (80/90). HPWMoV, however, was identified exclusively in central-eastern regions of Ukraine (Dnipropetrovsk, Donetsk, Zaporizhia, and Kharkiv) in 50% of the collected wheat samples (45/90). Similar to 2017, BSMV was never found (Table 2). Importantly, the screening of symptomatic wheat samples both for WSMV and HPWMoV allowed for assessing the occurrence of mono- and mixed viral infections inducing stripe mosaic in winter wheat. WSMV monoinfection was registered for 50% of symptomatic wheat plants (45/90), whereas only 11.1% of samples (10/90) exclusively contained HPWMoV in the absence of WSMV. Interestingly, 38.9% of the collected plant samples (35/90) were infected with a mix of WSMV and HPWMoV (Figure 2).

Further screening in 2019 confirmed WSMV circulation in every region probed (6/6), whereas HPWMoV was detected only in central-eastern parts of the country. The incidence of WSMV infection for symptomatic plants was 94.1% (80/85). In contrast to WSMV, HPWMoV was found in 29.4% of collected wheat samples (25/85). Similar to previous years, BSMV was not found. In 2019, 70.6% of wheat plants (60/85) were monoinfected by WSMV, 5.9% of wheat plants (5/85) were monoinfected by HPWMoV, while mixed infection (WSMV + HPWMoV) was registered for 23.5% (20/85) of collected plants (Figure 2).

**Figure 3:**
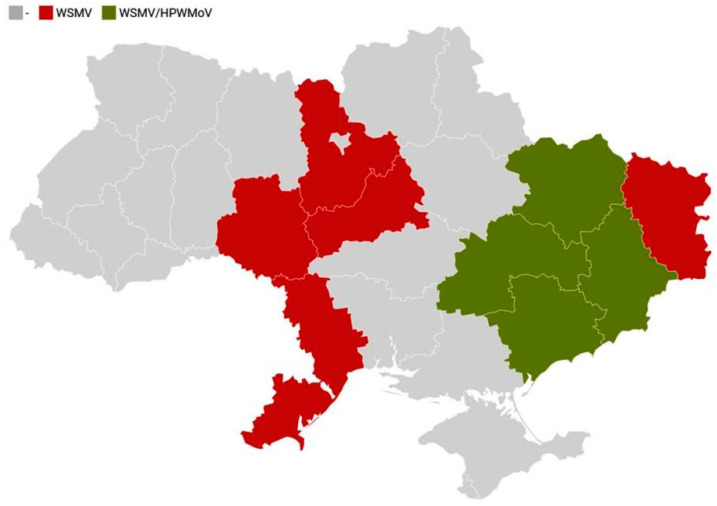
Schematic map of Ukraine showing regions where wheat streak mosaic virus (WSMV) only (red color) or WSMV and High Plains wheat mosaic virus (HPWMoV) (green color) were detected in this study. The map was generated using DataWrapper service (www.datawrapper.de, accessed on 1 April 2022).

Overall, a total of 245 plant samples showing virus-like symptoms (stripe mosaic, stunting) were collected in sampling sites in 2017–2019. On average, WSMV was detected by DAS-ELISA in 210/245 plant samples (85.7%), whereas HPWMoV was found in 70/175 plant samples (40%). BSMV was not detected in this work (Table 2). WSMV was found in every region tested (9/9), while HPWMoV was identified in four eastern regions of Ukraine but not in the central or southern parts (Figure 3).

In 2018–2019, the average incidence of WSMV monoinfection was 60% (105/175) of symptomatic wheat plants, HPWMoV monoinfection was registered for 8.6% (15/175) of collected samples, whereas mixed infection (WSMV + HPWMoV) was found in 31.4% of the samples (55/175).

Furthermore, the ELISA results obtained are in agreement that mixed infection of winter wheat plants by WSMV and HPWMoV may correlate with more severe visual symptoms exhibited with pronounced yellow stripe mosaic, shorter spikes, and plant stunting, as shown in Figure 1b.

Further, we analyzed winter wheat varieties of Ukrainian and German selections widely cultivated in Ukraine for their natural infection by WSMV and/or HPWMoV (Table 3).

Our results showed that 12/13 of the varieties tested were definitely susceptible to WSMV, but WSMV was not found in the Ukrainian variety Novosmuhlyanka in the field. Surprisingly, a similar study for HPWMoV showed that 53.8% (7/13) of these varieties were not naturally infected by this pathogen in Ukrainian agroecosystems. This list included five varieties of Ukrainian selection (Bohdana, Zymoyarka, Zolotokolosa, Misiya Odesska, and Smuhlyanka) and two varieties of German selection (Emil and Cubus).

### 3.2. Direct Virus Indication Using Electron Microscopy

**Figure 4:**
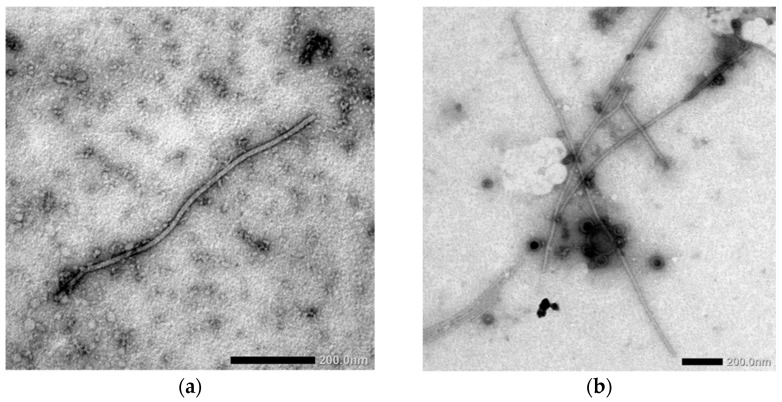
Morphology of viruses detected in sap of symptomatic winter wheat plants as detected by transmission electron microscopy: wheat streak mosaic virus (WSMV) monoinfection (**a**) and mixed infection of WSMV + High Plains wheat mosaic virus (HPWMoV) (**b**). Bars are indicated on images.

Virus presence and morphology in the partly clarified sap of symptomatic wheat plants was determined using transmission electron microscopy (Figure 4).

According to TEM data, filamentous viral particles ~700 nm in length and ~12–13 nm in diameter have been registered, in some cases accompanied by spherical double-membrane bodies ~90–110 nm in diameter. In all these cases, accurate morphological identification allowed us to attribute filamentous virions to potyviruses, whereas spherical particles were typical for emaraviruses.

### 3.3. Virus Detection by RT-PCR in ELISA-Positive Samples

Winter wheat samples previously shown to be positive for WSMV and/or HPWMoV in serological screening (see Table 2) were further subjected to virus identification using RT-PCR, also aiming at the amplification of a part of the coat protein gene (for WSMV), nucleoprotein gene (HPWMoV), or PIPO region (for TriMV) for subsequent phylogenetic analysis. In this molecular screening, we used oligonucleotide primers for the detection WSMV, HPWMoV, and TriMV as described in Table 1.

PCR products of the expected size for WSMV and HPWMoV were successfully amplified. However, TriMV was not identified in PCR-tested samples.

### 3.4. Phylogenetic Relationships and Sequence Similarity

Amplified subgenomic sequences of WSMV and HPWMoV were determined and deposited in DDBJ/EMBL/GenBank databases as indicated in Table S1. Overall, five partial nucleotide sequences of WSMV and three partial nucleotide sequences of HPWMoV were analyzed in this work.

The results indicated that these sequences corresponded to the respective parts of the genomes of WSMV (coat protein gene) and HPWMoV (nucleoprotein gene).

Pairwise comparison of nucleotide and amino acid sequences of the coat protein of five WSMV isolates used in this research showed that they were characterized by mutually high levels of identity (>97% at the nucleotide level). Moreover, these Ukrainian WSMV isolates demonstrated a high level of identity (>96.5% at the nucleotide level) with previously published wheat isolates from Ukraine (MW072786.1, MW072787.1, and MZ202336.1), Poland (MH939146.1, MH939145.1), Slovakia (EU723085.1, FJ613359.1, EU723086.1), Serbia (MT461299.1, MT461302.1), Russia (AF454459.1), Czech Republic (KY419569.1, KY419568.1), Italy (FJ606885.1), Germany (HG810954.1), France (HG810953.1), Lithuania (KJ720819.1), Hungary (MT780557.1), and Turkey (FJ606886.1), recovered mostly from wheat but also from barley and millet. WSMV isolates from other groups, A, C, and D, as well as those recovered from wild-growing grasses, and surprisingly, two other Ukrainian wheat WSMV isolates reported previously (MH523357.1 and MH523356.1), demonstrated a much lower degree of identity (~60–93% at the nucleotide level) to the five Ukrainian WSMV isolates used in this research, as shown in Table S2.

**Figure 5:**
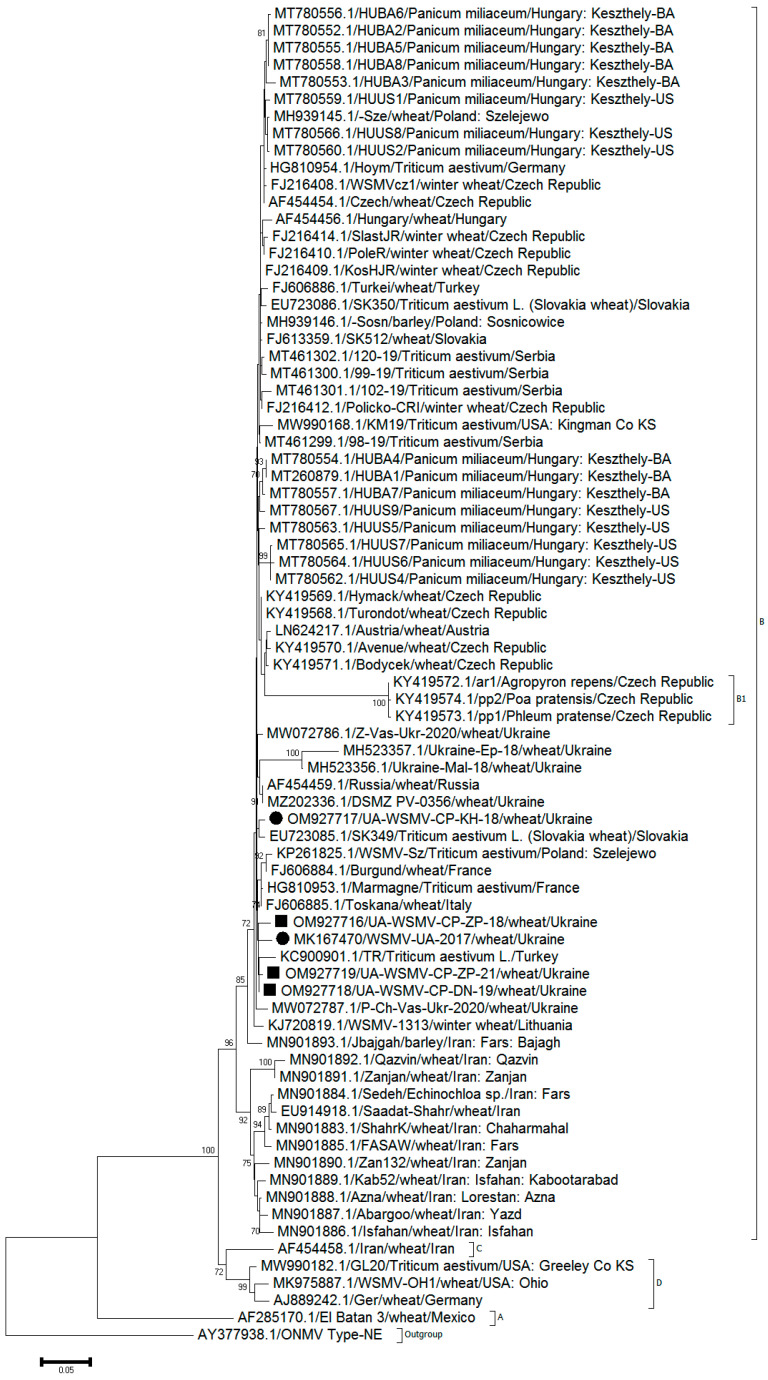
Neighbor-Joining (NJ) tree showing phylogenetic relationships between the isolates of wheat streak mosaic virus (WSMV). The coat protein gene region was used to construct the tree. Ukrainian isolates studied in this work are shown with dots (monoinfection of WSMV) and squares (mixed infection with HPWMoV). Bootstrap values <70 are not shown for respective branches. Oat necrotic mottle virus (ONMV) sequence (AY377938.1) was used as outgroup. Scale bar indicates 0.05 base substitutions/site.

For WSMV, NJ phylogenetic trees were initially calculated from the sequences of all isolates available from the GenBank. However, there were inconsistencies in, and poor bootstrap support for, some lineages in the resulting trees, as found previously for other potyviruses [50,51,52]. Therefore, the trees were recalculated from the partial genomes of 77 isolates using mostly WSMV isolates from group B. The relationships of these isolates were investigated by the NJ method (Figure 5).

The tree partitioned most of the sequences into the four consistent phylogenetic groups: A, B, C, and D. All the phylogenetic groupings were supported by high bootstrap values. As expected, all Ukrainian isolates (including five isolates described here and listed in Table S1) fell into group B in the NJ tree. Most of the sequences of WSMV including all five studied in this work were partitioned with a high degree of similarity whereas the other two isolates MH523357.1 and MH523356.1 were less similar.

Further, the nucleotide and amino acid sequence similarities between five WSMV isolates used in this work (Table S1) and five other Ukrainian isolates available from the GenBank were calculated (Table 4).

From Figure 5 and Table 4 and Table S2 it follows that the five studied WSMV isolates were highly similar to three previously described Ukrainian isolates, Z-Vas-Ukr-2020 (MW072786.1), P-Ch-Vas-Ukr-2020 (MW072787.1), and DSMZ_PV-0356 (MZ202336.1), but remained quite distinct from the other two isolates, Ukraine-Ep-18 (MH523357.1) and Ukraine-Mal-18 (MH523356.1), which were described earlier and, in turn, showed a high level of mutual identity.

Three HPWMoV sequences listed in Table S1 remain, for now, the only isolates of this virus originating from Ukraine. Pairwise comparison of nucleotide and amino acid sequences of part of the nucleoprotein of these three HPWMoV isolates showed that two wheat isolates (MK790263.1 and MK790262.1) were characterized by a high level of mutual identity (>98% at the nucleotide level). Strikingly, only the maize HPWMoV isolate (MK156130.1) was significantly less similar to these wheat isolates, demonstrating ~85% at the nucleotide level and just ~75–76% at the level of amino acids (Table S3).

When comparing other published HPWMoV sequences from around the world, wheat Ukrainian HPWMoV isolates were most similar (~88–90% of identity at the nucleotide level) to other wheat isolates originating from the USA (MT762120.1, MN250347.1, KT995102.1, KT988889.1, KT988882.1, KT988881.1, KT970501.1, and DQ324466.1). Surprisingly, despite the maize HPWMoV isolate from Ukraine being less related to other published sequences, it was also more similar to wheat isolates from the USA listed above, with MT563400.1 being the best hit, showing only ~83% of identity at the nucleotide level.

**Figure 6:**
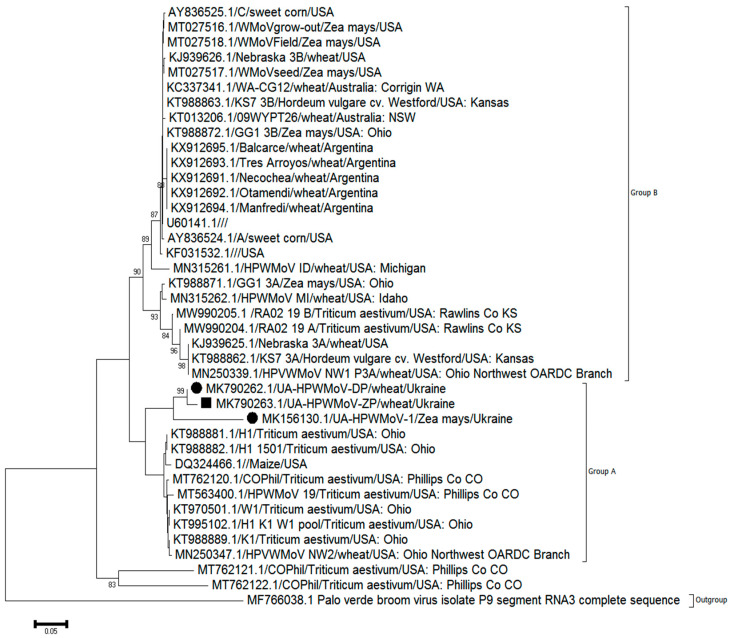
Neighbor-Joining (NJ) tree showing phylogenetic relationships between the isolates of High Plains wheat mosaic virus (HPWMoV). The nucleoprotein gene region was used to construct the tree. Ukrainian isolates studied in this work are shown with dots (monoinfection of HPWMoV) and squares (mixed infection with wheat streak mosaic virus (WSMV)). Bootstrap values <70 are not shown for respective branches. Palo verde broom virus sequence (MF7666038/1) was used as outgroup. Scale bar indicates 0.05 base substitutions/site.

For HPWMoV, an NJ phylogenetic tree was calculated using sequences of all 39 isolates available from the GenBank (Figure 6).

Most of the HPWMoV sequences were partitioned into two phylogenetic groups, A and B, as supported by high bootstrap values. All three Ukrainian isolates described here and listed above in Tables S1 and S3 fell into group A, which was originally nearly exclusively represented by wheat isolates recovered from the USA. On the NJ tree, Ukrainian isolates collected from wheat (MK790262 and MK790263) demonstrated a high degree of similarity (>98% at the nucleotide level) and formed a separate cluster with the somewhat less similar (>85% at the nucleotide level) HPWMoV isolate recovered from maize (MK156130).

## 4. Discussion

### 4.1. Occurrence of WSMV and HPWMoV

In the present study, WSMV was confirmed to be a predominant virus in every screened region in the eastern, southern, and central parts of Ukraine (Luhansk, Donetsk, Kharkiv, Kyiv, Cherkassy, Vinnytsia, Dnipropetrovsk, Zaporizhia, and Odessa regions) (Figure 3, Table 2), where it caused leaf streaking mosaic and plant stunting (Figure 1) in over 85% of symptomatic wheat plants. This is in agreement with previous data [33,34] and also expands current knowledge on WSMV spread in Ukraine. Despite the fact screening of other parts (especially western regions) of Ukraine for WSMV was not conducted in this work, wide cereal cultivation in Ukraine and domestic transportation of seed material, as well as international seed trade coupled with mechanical and seed transmission of this virus, do not leave many doubts that WSMV is presumably ubiquitous in Ukraine.

Complementing results of serological screening, direct indication, and molecular detection (Figure 2 and Figure 4, and Table 2) proved the wide spread of HPWMoV in the central-eastern part of Ukraine (Dnipropetrovsk, Donetsk, Zaporizhia, and Kharkiv regions) with a 40% overall incidence rate. However, HPWMoV was not identified in the remaining screened regions where WSMV was abundant. This is in agreement with our previous report on HPWMoV occurrence in Ukraine [35].

The significant spread of WSMV and HPWMoV and their dependence on a unique vector, wheat curl mite, indicates the importance of vector monitoring and control for the successful management of these viral diseases in Ukraine. For decades, *A. tosichella* was mostly found in the eastern part of the country where the climatic conditions (i.e., average annual temperature, etc.) may have favored its spread and overwintering, similar to other parts of the world [53,54] The current data on the occurrence of this virus vector in other regions of Ukraine are to be elucidated.

### 4.2. Patterns of Mono- and Mixed Infection of Winter Wheat with WSMV and HPWMoV

Interestingly, as much as 60% of symptomatic plants were monoinfected with WSMV, whereas wheat monoinfection by HPWMoV was registered for only ~9% of plants. In line with the overall virus spread, this suggests prolonged circulation of WSMV in Ukrainian agroecosystems but the presumably recent introduction of HPWMoV. In contrast to the relatively small number of HPWMoV-moninfected plants, over 31% of wheat plants were mixed infected with both viruses, which correlated with more severe visual symptoms (Figure 1b). The wide occurrence of WMSV/HPWMoV mixed infection was also shown, for example, in the USA, where HPWMoV typically occurs along with WSMV, because both viruses are transmitted by the same vector [55]. In this context, a large portion of mixed infected wheat plants (>30%) may suggest that this pattern of infection may favor a more efficient spread/transmission of HPWMoV by the vector when compared to its monoinfection (<9%). However, this will require more research on virus–vector relationships. If proven, a wider spread of HPWMoV in Ukraine is to be expected in the coming years in the regions where the wheat curl mite (Figure 1c) is present and susceptible cereals are commonly cultivated. In this view, it is worth mentioning that HPWMoV may be transmitted not only by its vector but also by maize seed [29]. The discovery of HPWMoV isolates on both wheat and maize in Ukraine may suggest that this virus was either introduced with seed material with subsequent initial circulation on maize or that such virus introduction into Ukraine has occurred several times. Moreover, the possibility of the introduction of HPWMoV-contaminated mites and/or infected plant material cannot be ruled out.

### 4.3. Susceptibility of Different Varieties of Winter Wheat to WSMV and HPWMoV Infections

The screening of 13 commonly cultivated wheat varieties of Ukrainian and German selection confirmed that all but one variety were naturally infected with WSMV in field conditions. Symptomatic samples of the Novosmuhlyanka variety were not WSMV-infected (but, in turn, were HPWMoV-positive). This piece of data indicates that all such varieties are not only susceptible to WSMV but also confer the survival of its vector [56], underpinning a need for careful reassessment of the choice of cultivars when it comes to winter wheat cultivation in Ukraine.

On the contrary, seven winter wheat varieties (Bohdana, Zymoyarka, Zolotokolosa, Misiya, Odesska, Smuhlyanka, Emil, and Cubus—the latter two being of German selection) were not HPWMoV-infected in the field (Table 3). These varieties may therefore represent potential candidates for further exploration via challenging experiments in a search for possible sources of tolerance/insusceptibility/resistance of wheat plants to this pathogen.

### 4.4. Phylogenetic Analysis of WSMV and HPWMoV Isolates from Ukraine

The simplest interpretation of the phylogenetic analyses is that five WSMV isolates identified and studied in this work expectedly belonged to group B and were characterized by a high degree of similarity among themselves and to many other known isolates of this virus found in Ukraine and in neighboring or even more distant European countries (Poland, Czech Republic, Slovakia, Russia, Hungary, Italy, Germany, France, Turkey, etc.). The line of evidence suggests high overall homogeneity of WSMV population(s) circulating in screened regions of Ukraine and Europe. However, two WSMV isolates (MH523357.1 and MH523356.1) found in the Kharkiv region (east of Ukraine) some time ago (Mishchenko et al., 2019) were far more distant from the five WSMV isolates described in this study. This is indicative of the tentative occurrence of several lineages of WSMV circulating in Ukraine. Wide-scale populational research with full genomic sequencing of WSMV isolates will aid in establishing their possible origins.

As for HPWMoV, the obtained data were in agreement with our previous results showing high homogeneity of two wheat isolates with a somewhat smaller degree of identity typical for the only maize HPWMoV isolate found until now. All these isolates, however, were most similar to the known sequences of this virus originating from the USA, suggesting the possible introduction of HPWMoV into Ukraine with seed/plant material, which may have occurred a number of times. Despite the currently limited geographical spread of HPWMoV in Ukraine, its occurrence in two different and widely cultivated cereal crops may pose a risk of consequential virus distribution into ‘new’ niches, following the footsteps of WSMV and their common vector.

Interestingly, we were not able to trace any notable phylogenetic differences for WSMV and HPWMoV found in either mono- or mixed infection in winter wheat collected from various regions at different years. As shown in Figure 5, WSMV sequences recovered from mono- (WSMV-UA-2017 (MK167470), UA-WSMV-CP-KH-18 (OM927717)) or mixed infected (UA-WSMV-CP-ZP-18 (OM927716), UA-WSMV-CP-DN-19 (OM927718), and UA-WSMV-CP-ZP-21 (OM927719)) samples were highly similar (>97%) regardless of the pattern of infection. A similar tendency was demonstrated for a smaller number of HPWMoV isolates (Figure 6), where UA-HPWMoV-DP (MK790262.1) and UA-HPWMoV-ZP (MK790263.1) sequences recovered from mono- and mixed infected winter wheat, respectively, were over 98% identical. Despite the partial sequencing and relatively small number of virus isolates found in this research, these data are confirmative of the current absence of the effect of a second virus on the replication of the initial one in terms of WSMV/HPWMoV complex in wheat in Ukraine.

In summary, although an added understanding of WSMV and HPWMoV biology, epidemiology, and phylogeny was brought about with this study, the wide survey of these pathogens and their vector in different (mostly southern and western) regions of Ukraine is necessary to confirm our conclusions. However, to our knowledge, the present study shows the occurrence of wheat streak mosaic virus in mono- and mixed infection with High Plains wheat mosaic virus in winter wheat in Ukraine and describes the evolutionary relationships between Ukrainian isolates and the worldwide isolates of these pathogens previously reported.

## 5. Conclusions

Complementing results of visual and serological screening, direct indication, and molecular detection prove the wide spread of WSMV (>85% incidence rate) and HPWMoV (40% incidence rate) in the central-eastern part of Ukraine (Dnipropetrovsk, Donetsk, Zaporizhia, and Kharkiv regions) where these two pathogens may successfully survive in the form of mono- or mixed infections.

In Ukraine, WSMV more frequently induces monoinfection (60%), whereas HPWMoV is typically found in mixed infection with WSMV (>31%) but not in monoinfection (<9%).

Nearly all winter wheat varieties commonly cultivated in Ukraine and screened for natural virus infection are susceptible to WSMV. In contrast, 53% of these varieties (7/13) were not naturally infected with HPWMoV, possibly representing candidates for sources of resistance.

Five WSMV isolates found in this research are characterized by high mutual identity (>97% at the nucleotide level) and high identity (>96.5% at the nucleotide level) to previously published wheat isolates from Ukraine and many other European countries. In turn, the three HPWMoV isolates described here are most similar (>85–98% at the nucleotide level) to the USA isolates of this virus found earlier.

## Figures and Tables

**Table 1:** Sequences of oligonucleotide primers used in this work.

Virus	Gene/Region	Forward Primer	Reverse Primer	Expected Product Length	Reference
WSMV	Coat protein	GAG AGC AAT ACT GCG TGT ACG	GCA TAA TGG CTC GAA GTG ATG	750 bp	[39]
HPWMoV	Nucleoprotein	TTT ATG GCT CTT TGT ATT GG	TAT GTT TCC CCT CTT TGT G	339 bp	[40]
TriMV	PIPO	CTT AAG CAC ATG TTA CAA TC	GTC CCT GAT AAC TAA TTC TA	1200 bp	[41]

WSMV—wheat streak mosaic virus, HPWMoV—High Plains wheat mosaic virus, TriMV—triticum mosaic virus.

**Table 2:** Detection of WSMV, BSMV, and HPWMoV by DAS-ELISA in symptomatic winter wheat samples collected in 2017–2019 (mixed infection is shown in bold).

Region	District	Year of Sampling	BSMV *	WSMV *	HPWMoV *
Vinnytsia	Bershadskiy	2017	0/5	5/5	-
Dnipropetrovsk	Petropavlivskiy	2017	0/15	15/15	-
	Pokrovskiy	2017	0/10	10/10	-
Donetsk	Maryinskiy	2017	0/5	0/5	-
Kyiv	Kyiv-Svyatoshinskiy	2017	0/10	5/10	-
Luhansk	Novoaidarskiy	2017	0/5	5/5	-
Odessa	Ovidiopolskiy	2017	0/10	5/10	-
Kharkiv	Chuguyivskiy	2017	0/10	5/10	-
Vinnytsia	Bershadskiy	2018	0/5	5/5	0/5
Dnipropetrovsk	Mezhivskiy	2018	0/5	5/5 **	5/5 **
	Pokrovskiy	2018	0/5	0/5	5/5
Donetsk	Oleksandrivskiy	2018	0/15	10/15 **	15/15 **
	Pokrovskiy	2018	0/5	5/5 **	5/5 **
Zaporizhia	Vasylivskiy	2018	0/10	10/10 **	10/10 **
Kyiv	Kyiv-Svyatoshinskiy	2018	0/10	10/10	0/10
Kharkiv	Kupyanskiy	2018	0/20	20/20 **	5/20 **
	Kharkivskiy	2018	0/15	15/15	0/15
Vinnytsia	Bershadskiy	2019	0/10	10/10	0/10
Dnipropetrovsk	Mezhivskiy	2019	0/30	25/30 **	20/30 **
	Pavlogradskiy	2019	0/5	5/5	0/5
	Synelnykivskiy	2019	0/5	5/5	0/5
Zaporizhia	Bilmatskiy	2019	0/5	5/5	0/5
	Rozivskiy	2019	0/5	5/5	0/5
Kyiv	Kyiv-Svyatoshinskiy	2019	0/5	5/5	0/5
Kharkiv	Pechenizkiy	2019	0/15	15/15 **	5/15 **
Cherkassy	Umanskiy	2019	0/5	5/5	0/5
**Total**			0/245	210/245	70/175
**Percentage of infected plants**			0	85.7	40

BSMV—barley stripe mosaic virus, WSMV—wheat streak mosaic virus, HPWMoV—High Plains wheat mosaic virus. (*) positive samples/collected samples. (-) not tested. (**) mixed WSMV/HPWMoV infection.

**Table 3:** Occurrence of natural infection of WSMV and HPWMoV in Ukrainian and German varieties of winter wheat cultivated in Ukraine.

Cultivar	Country of Origin of Cultivar	Virus Infection *	
		WSMV	HPWMoV
Novosmuhlyanka	Ukraine	−	+
Garantiya Odesska	Ukraine	+	+
Donetska 48	Ukraine	+	+
Dostatok	Ukraine	+	+
Podolyanka	Ukraine	+	+
Bohdana	Ukraine	+	−
Zymoyarka	Ukraine	+	−
Zolotokolosa	Ukraine	+	−
Misiya Odesska	Ukraine	+	−
Smuhlyanka	Ukraine	+	−
Emil	Germany	+	−
Cubus	Germany	+	−
Skagen	Germany	+	+

WSMV—wheat streak mosaic virus, HPWMoV—High Plains wheat mosaic virus. * (+)—naturally infected plants, (−)—plants not infected naturally.

**Table 4:** Pairwise distances of nucleotide (below diagonal) and amino acid (above diagonal) partial sequences of Ukrainian isolates of WSMV. WSMV isolates described in this study are shown in bold.

Isolate Name (GenBank Accession Number)	WSMV-UA-2017 (MK167470)	UA-WSMV-CP-ZP-18 (OM927716)	UA-WSMV-CP-ZP-21 (OM927719)	UA-WSMV-CP-DN-19 (OM927718)	UA-WSMV-CP-KH-18 (OM927717)	P-Ch-Vas-Ukr-2020 (MW072787.1)	Ukraine-Ep-18 (MH523357.1)	Ukraine-Mal-18 (MH523356.1)	Z-Vas-Ukr-2020 (MW072786.1)	DSMZ_PV-0356 (MZ202336.1)
**WSMV-UA-2017** **(MK167470)**	-	0.022	0.016	0.011	0.021	0.021	0.142	0.074	0.016	0.016
**UA-WSMV-CP-ZP-18** **(OM927716)**	0.026	-	0.016	0.011	0.011	0.017	0.135	0.064	0.005	0.005
**UA-WSMV-CP-ZP-21** **(OM927719)**	0.017	0.020	-	0.005	0.016	0.021	0.124	0.068	0.011	0.011
**UA-WSMV-CP-DN-19** **(OM927718)**	0.013	0.016	0.004	-	0.011	0.016	0.130	0.062	0.005	0.005
**UA-WSMV-CP-KH-18** **(OM927717)**	0.023	0.022	0.013	0.010	-	0.016	0.130	0.062	0.005	0.005
P-Ch-Vas-Ukr-2020 (MW072787.1)	0.027	0.026	0.017	0.013	0.019	-	0.137	0.068	0.011	0.011
Ukraine-Ep-18 (MH523357.1)	0.100	0.102	0.087	0.087	0.093	0.098	-	0.062	0.124	0.124
Ukraine-Mal-18 (MH523356.1)	0.064	0.063	0.053	0.049	0.055	0.060	0.039	-	0.056	0.056
Z-Vas-Ukr-2020 (MW072786.1)	0.021	0.020	0.012	0.008	0.013	0.017	0.087	0.049	-	0.000
DSMZ_PV-0356 (MZ202336.1)	0.021	0.020	0.012	0.008	0.013	0.017	0.087	0.045	0.012	-

WSMV—wheat streak mosaic virus.

## Data Availability

All published data are available on request. All accession numbers for sequences of WSMV and HPWMoV generated in this study are correct. MK167470 is available already, while OM927716, OM927719, OM927718, and OM927717 will become available upon publication of this paper or by February 2032.

## Supplementary Materials

The following supporting information can be downloaded at: https://www.mdpi.com/article/10.3390/v14061220/s1, Table S1: List of sequences of Ukrainian isolates of WSMV and HPWMoV recovered and analyzed in this work; Table S2: Nucleotide and amino acid identity of WSMV isolates described in this research (shown in bold) with other WSMV isolates; Table S3: Nucleotide and amino acid identity of HPWMoV isolates described in this research (shown in bold) with other HPWMoV isolates.
