# Supplementary material for: Occurrence and Characterization of Wheat Streak Mosaic Virus Found in Mono- and Mixed Infection with High Plains Wheat Mosaic Virus in Winter Wheat in Ukraine
Source: Viruses. 2022 Jun 3;14(6):1220. doi: 10.3390/v14061220 (PMC9229632; doi:10.3390/v14061220)
Supplement: Supplementary file 1 [file viruses-14-01220-s001.zip › viruses-1735495-supplementary.pdf]

Table S1. List of sequences of Ukrainian isolates of WSMV and HPWMoV recovered and analyzed in this work.

GenBank Accession number	Virus	Gene	Isolate name	Year of sampling	Region of sampling
MK167470	WSMV	Coat protein	WSMV-UA-2017	2017	Kyiv
OM927716	WSMV	Coat protein	UA-WSMV-CP-ZP-18	2018	Zaporizhia
OM927717	WSMV	Coat protein	UA-WSMV-CP-KH-18	2018	Kharkiv
OM927718	WSMV	Coat protein	UA-WSMV-CP-DN-19	2019	Dnipropetrovsk
OM927719	WSMV	Coat protein	UA-WSMV-CP-ZP-21	2021	Zaporizhia
MK790262	HPWMoV	Nucleoprotein	UA-HPWMoV-DP	2018	Dnipropetrovsk
MK790263	HPWMoV	Nucleoprotein	UA-HPWMoV-ZP	2018	Zaporizhia
MK156130	HPWMoV	Nucleoprotein	UA-HPWMoV-1	2018	Vinnytsia (maize)

Table S2. Nucleotide and amino acid identity of WSMV isolates described in this research (shown in bold) with other WSMV isolates

GenBank Accession number	Isolate name	Country of origin	Nucleic acid identity, %					Amino acid identity, %				
			WSMV-UA-2017	UA-WSMV-CP-ZP-18	UA-WSMV-CP-ZP-21	UA-WSMV-CP-DN-19	UA-WSMV-CP-KH-18	WSMV-UA-2017	UA-WSMV-CP-ZP-18	UA-WSMV-CP-ZP-21	UA-WSMV-CP-DN-19	UA-WSMV-CP-KH-18
**MK167470**	**WSMV-UA-2017**	**Ukraine**	**-**					**-**				
**OM927716**	**UA-WSMV-CP-ZP-18**	**Ukraine**	**97,4**	**-**				**97,8**	**-**			
**OM927719**	**UA-WSMV-CP-ZP-21**	**Ukraine**	**98,3**	**98**	**-**			**98,4**	**98,3**	**-**		
**OM927718**	**UA-WSMV-CP-DN-19**	**Ukraine**	**98,7**	**98,4**	**99,6**	**-**		**98,9**	**98,9**	**99,5**	**-**	
**OM927717**	**UA-WSMV-CP-KH-18**	**Ukraine**	**97,7**	**97,8**	**98,7**	**99**	**-**	**97,9**	**98,9**	**98,4**	**98,9**	**-**
MW072786.1	Z-Vas-Ukr-2020	Ukraine	97,9	98	98,8	99,2	98,7	98,4	99,4	98,9	99,5	99,5
MW072787.1	P-Ch-Vas-Ukr-2020	Ukraine	97,3	97,4	98,3	98,7	98,1	97,8	98,3	97,8	98,4	98,4
MH523356.1	Ukraine-Mal-18	Ukraine	93,6	93,7	94,7	95,1	94,5	92,6	93,5	93,2	93,8	93,7
MH523357.1	Ukraine-Ep-18	Ukraine	90	89,8	91,3	91,3	90,7	85,6	86,4	87,5	86,9	86,9
MZ202336.1	DSMZ_PV-0356	Ukraine	97,9	98	98,8	99,2	98,7	98,4	99,4	98,9	99,5	99,5
MW990168.1	KM19	USA: Kingman Co_KS	96,5	97	97,5	97,9	97,3	97,3	98,3	97,9	98,4	98,4
MW990182.1	GL20	USA: Greeley Co_KS	89,9	89,9	91	91,2	90,6	97,3	98,3	97,9	98,4	98,4
MT780567.1	HUUS9	Hungary: Keszthely-US	97,5	97,6	98,5	98,8	98,3	98,4	99,4	98,9	99,5	99,5
MT780566.1	HUUS8	Hungary: Keszthely-US	97,3	97,4	98,3	98,6	98,1	98,4	99,4	98,9	99,5	99,5
MT780565.1	HUUS7	Hungary: Keszthely-US	97,1	97	97,9	98,3	97,7	97,3	98,3	97,9	98,4	98,4
MT780564.1	HUUS6	Hungary: Keszthely-US	96,9	96,8	97,7	98,1	97,5	97,3	98,3	97,9	98,4	98,4
MT780563.1	HUUS5	Hungary: Keszthely-US	97,7	97,8	98,7	99	98,5	98,4	99,4	98,9	99,5	99,5
MT780562.1	HUUS4	Hungary: Keszthely-US	97,3	97,2	98,1	98,5	97,9	97,9	98,9	98,4	98,9	98,9
MT780560.1	HUUS2	Hungary: Keszthely-US	97,1	97,2	98,1	98,5	97,9	98,4	99,4	98,9	99,5	99,5
MT780559.1	HUUS1	Hungary: Keszthely-US	97,1	97,2	98,1	98,5	97,9	98,4	99,4	98,9	99,5	99,5
MT780558.1	HUBA8	Hungary: Keszthely-BA	97,3	97,4	98,3	98,6	98,1	98,4	99,4	98,9	99,5	99,5
MT780557.1	HUBA7	Hungary: Keszthely-BA	97,7	97,8	98,7	99	98,5	98,4	99,4	98,9	99,5	99,5
MT780556.1	HUBA6	Hungary: Keszthely-BA	97,1	97,2	98,1	98,5	97,9	98,4	99,4	98,9	99,5	99,5
MT780555.1	HUBA5	Hungary: Keszthely-BA	97,3	97,4	98,3	98,6	98,1	98,4	99,4	98,9	99,5	99,5
MT780554.1	HUBA4	Hungary: Keszthely-BA	97,5	97,6	98,5	98,8	98,3	98,4	99,4	98,9	99,5	99,5
MT780553.1	HUBA3	Hungary: Keszthely-BA	96,5	96,6	97,5	97,9	97,3	97,3	98,3	97,9	98,4	98,4
MT780552.1	HUBA2	Hungary: Keszthely-BA	97,3	97,4	98,3	98,6	98,1	98,4	99,4	98,9	99,5	99,5
MT461302.1	120-19	Serbia	97,7	98	98,7	99	98,5	97,9	99,4	98,4	98,9	98,9

MT461301.1	102-19	Serbia	96,9	97	97,9	98,3	97,7	98,4	99,4	98,9	99,5	99,5
MT461300.1	99-19	Serbia	97,5	98,2	98,5	98,8	98,3	97,9	99,4	98,4	98,9	98,9
MT461299.1	98-19	Serbia	97,9	98	98,8	99,2	98,7	98,4	99,4	98,9	99,5	99,5
MT260879.1	HUBA1	Hungary: Keszthely-BA	97,5	97,6	98,5	98,8	98,3	98,4	99,4	98,9	99,5	99,5
MN901893.1	Jbajgah	Iran:_Fars:_Bajagh	96,3	96	96,9	97,3	96,7	98,4	99,4	98,9	99,5	99,5
MN901892.1	Qazvin	Iran: Qazvin, Qazvin	91,7	91,4	92,3	92,5	92,3	98,4	99,4	98,9	99,5	99,5
MN901891.1	Zanjan	Iran: Zanjan, Zanjan	92,7	92,6	93,4	93,6	93,4	98,4	99,4	98,9	99,5	99,5
MN901890.1	Zan132	Iran: Zanjan, Zanjan	93,6	93,5	93,8	94,2	94	98,4	99,4	98,9	99,5	99,5
MN901889.1	Kab52	Iran: Isfahan: Kabootarabad	93,4	93,4	94,2	94,6	94,4	98,4	99,4	98,9	99,5	99,5
MN901888.1	Azna	Iran:_Lorestan:_Azna	93,8	93,9	94,6	95,1	94,8	98,4	99,4	98,9	99,5	99,5
MN901887.1	Abargoo	Iran: Yazd, Abargoo	93,2	93,2	94	94,4	94,2	97,9	98,9	98,4	98,9	98,9
MN901886.1	Isfahan	Iran: Isfahan, Isfahan	93	92,8	93,6	93,8	93,6	97,3	98,3	97,9	98,4	98,4
MN901885.1	FASAW	Iran: Fars, Fasa	93,2	93,7	93,6	94	93,8	98,4	99,4	98,9	99,5	99,5
MN901884.1	Sedeh	Iran: Fars, Sedeh	92,5	93	93,4	93,8	93,6	98,4	99,4	98,9	99,5	99,5
MN901883.1	ShahrK	Iran: Chaharmahal, Shahrekord	92,8	93,2	93,6	94	93,8	97,9	98,9	98,4	98,9	98,9
MK975887.1	WSMV-OH1	USA: Ohio	89	89	89,7	89,9	89,2	96,2	97,2	96,8	97,3	97,3
MH939146.1	-Sosn	Poland: Sosnicowice	97,9	98	98,8	99,2	98,7	98,4	99,4	98,9	99,5	99,5
MH939145.1	-Sze	Poland: Szelejewo	97,1	97,2	98,1	98,5	97,9	98,4	99,4	98,9	99,5	99,5
LN624217.1	Austria	Austria	97,1	97,4	98,1	98,5	97,9	97,3	98,3	97,9	98,4	98,4
KY419574.1	pp2	Czech Republic	84,4	84,7	85,5	86	85,5	78	78	78,1	78,7	78,6
KY419573.1	pp1	Czech Republic	84,4	84,7	85,5	86	85,5	78	77,9	78	78,7	78,6
KY419572.1	ar1	Czech Republic	84,6	85	85,8	86,2	85,8	78,6	78,6	78,7	79,3	79,3
KY419571.1	Bodycek	Czech Republic	97,3	97,4	98,3	98,7	98,1	97,8	98,9	98,4	98,9	98,9
KY419570.1	Avenue	Czech Republic	97,1	97,2	98,1	98,5	97,9	96,8	97,8	97,3	97,9	97,8
KY419569.1	Hymack	Czech Republic	98,1	98,2	99	99,4	98,8	98,4	99,4	98,9	99,5	99,5
KY419568.1	Turondot	Czech Republic	98,1	98,2	99	99,4	98,8	98,4	99,4	98,9	99,5	99,5
KP261825.1	WSMV-Sz	Poland: Szelejewo	97,3	97,4	98,1	98,3	97,7	97,3	98,3	97,9	98,4	98,4
KJ720819.1	WSMV-1313	Lithuania	97,5	97,6	98,1	98,5	97,9	97,9	98,9	98,4	98,9	98,9
KC900901.1	TR	Turkey	96,9	97	97,9	98,3	97,3	98,9	98,9	99,5	100	98,9
HG810954.1	Hoym	Germany	97,5	97,6	98,5	98,8	98,3	98,4	99,4	98,9	99,5	99,5
HG810953.1	Marmagne	France	97,9	98	99	99,2	98,7	98,4	99,4	98,9	99,5	99,5
FJ613359.1	SK512	Slovakia	97,9	98	98,8	99,2	98,7	97,9	98,9	98,4	98,9	98,9
FJ606886.1	Turkei	Turkey	97,3	97,4	98,3	98,7	98,1	98,4	99,4	98,9	99,5	99,5
FJ606885.1	Toskana	Italy	98,1	98,2	99,2	99,4	98,8	98,4	99,4	98,9	99,5	99,5
FJ606884.1	Burgund	France	97,9	98	98,7	98,8	98,3	98,4	99,4	98,9	99,5	99,5
FJ216414.1	SlastJR	Czech Republic	97,3	97,4	98,3	98,7	98,1	97,9	98,9	98,4	98,9	98,9
FJ216412.1	Policko-CRI	Czech Republic	97,9	98	98,8	99,2	98,7	98,4	99,4	98,9	99,5	99,5
FJ216410.1	PoleR	Czech Republic	97,7	97,8	98,7	99	98,5	98,4	99,4	98,9	99,5	99,5
FJ216409.1	KosHJR	Czech Republic	98,1	98,2	99	99,4	98,8	98,4	99,4	98,9	99,5	99,5
FJ216408.1	WSMVcz1	Czech Republic	97,5	97,6	98,5	98,8	98,3	97,9	98,9	98,4	98,9	98,9
EU914918.1	Saadat-Shahr	Iran	92,1	92,6	93,2	93,4	93,2	98,4	99,4	98,9	99,5	99,5
EU723086.1	SK350	Slovakia	97,5	97,6	98,5	98,8	98,3	98,4	99,4	98,9	99,5	99,5
EU723085.1	SK349	Slovakia	98,1	97,8	98,7	99	98,8	98,4	99,4	98,9	99,5	99,5
AF454459.1	Russia	Russia	97,9	98	98,8	99,2	98,7	98,4	99,4	98,9	99,5	99,5
AJ889242.1	Ger	Germany	89,3	88,3	89,9	90,2	89	97,3	96,1	96,8	97,3	96,2
AF454458.1	Iran	Iran	88,8	88,1	89,5	89,7	89	97,3	98,3	97,9	98,4	98,4
AF285170.1	El Batan 3	Mexico	68,8	69,5	69,1	69,7	68,8	81	81,1	81,7	82,3	81,6
AF454456.1	Hungary	Hungary	97,3	97	98,3	98,7	97,7	97,9	98,3	98,4	98,9	98,4
AF454454.1	Czech	Czech Republic	97,7	97,8	98,7	99	98,5	98,4	99,4	98,9	99,5	99,5

Table S3. Nucleotide and amino acid identity of HPWMoV isolates described in this research (shown in bold) with other HPWMoV isolates.

GenBank Accession number	Isolate name	Country of origin	Nucleic acid identity, %			Amino acid identity, %		
			UA-HPWMoV-ZP	UA-HPWMoV-DP	UA-HPWMoV-1	UA-HPWMoV-ZP	UA-HPWMoV-DP	UA-HPWMoV-1
**MK790263.1**	**UA-HPWMoV-ZP**	**Ukraine**	**-**			**-**		
**MK790262.1**	**UA-HPWMoV-DP**	**Ukraine**	**98,33**	**-**		**95,9**	**-**	
**MK156130.1**	**UA-HPWMoV-1**	**Ukraine**	**85,32**	**86,55**	**-**	**75,5**	**76,64**	**-**
U60141.1			84,41	86,05	76	84,7	87,94	72,8
MW990205.1	RA02 19 B	USA: Rawlins Co KS	82,74	84,41	75,3	83,4	86,7	70,76
MW990204.1	RA02 19 A	USA: Rawlins Co KS	82,36	84,04	74,6	84,5	87,79	70,76
MT762122.1	COPhil	USA: Phillips Co CO	66,02	66,11	63,8	56,1	57,04	48,88
MT762121.1	COPhil	USA: Phillips Co CO	69,05	69,14	62,9	63,2	64,07	58,13
MT762120.1	COPhil	USA: Phillips Co CO	88,53	90,07	80,1	88,1	91,3	74,49
MT563400.1	HPWMoV 19	USA: Phillips Co CO	80,43	83,81	83,1	87,1	91,13	90,64
MT027518.1	WMoVField	USA	83,75	85,23	76	84,9	87,36	72,8
MT027517.1	WMoVseed	USA	85,5	86,68	74,8	84,7	87,77	66,68
MT027516.1	WMoVgrow-out	USA	83,46	84,97	76	84,7	87,2	72,8
MN315262.1	HPWMoV MI	USA: Idaho	83,64	85,29	77,4	85,7	88,93	72,8
MN315261.1	HPWMoV ID	USA: Michigan	82,67	84,35	73,8	83,5	86,74	70,51
MN250347.1	HPVWMoV NW2	USA: Ohio Northwest OARDC Branch	88,12	89,67	80,1	86,9	90,12	74,49
MN250339.1	HPVWMoV NW1 P3A	USA: Ohio Northwest OARDC Branch	81,89	83,58	74,5	84,5	87,73	70,51
KX912695.1	Balcarce	Argentina	83,81	85,52	76,7	84,1	87,43	72,8
KX912694.1	Manfredi	Argentina	83,56	85,29	76,7	84	87,39	72,8
KX912693.1	Tres Arroyos	Argentina	83,87	85,57	76,7	84,2	87,5	72,8
KX912692.1	Otamendi	Argentina	83,49	85,23	76,7	84	87,39	72,8
KX912691.1	Necochea	Argentina	83,75	85,46	76,7	84,1	87,43	72,8
KT995102.1	H1 K1 W1 pool	USA: Ohio	88,91	90,44	80,1	86,9	90,12	74,49
KT988889.1	K1	USA: Ohio	88,52	90,06	80,1	86,9	90,12	74,49
KT988882.1	H1 1501	USA: Ohio	89,31	90,83	80,8	86,9	90,12	74,49
KT988881.1	H1	USA: Ohio	89,31	90,83	80,8	86,9	90,12	74,49
KT988872.1	GG1 3B	USA: Ohio	84,41	86,05	76	84,7	87,94	72,8
KT988871.1	GG1 3A	USA: Ohio	83,89	85,55	76,3	85,3	88,62	71,46
KT988863.1	KS7 3B	USA: Kansas	84,41	86,05	76	84,7	87,94	72,8
KT988862.1	KS7 3A	USA: Kansas	81,89	83,58	74,5	84,5	87,73	70,51
KT970501.1	W1	USA: Ohio	88,91	90,44	80,1	86,9	90,12	74,49
KT013206.1	09WYPT26	Australia: NSW	82,69	84,48	75,3	83,7	87,1	72,8
KJ939626.1	Nebraska 3B	USA	83,98	85,63	75,3	83,5	86,78	70,64
KJ939625.1	Nebraska 3A	USA	81,89	83,58	74,5	84,5	87,73	70,51
KF031532.1		USA	82,77	84,52	74,6	84,1	87,43	72,8
KC337341.1	WA-CG12	Australia: Corrigin WA	83,16	84,94	76	83,7	87,1	72,8
DQ324466.1		USA	88,14	89,68	80,8	86,9	90,12	74,49
AY836525.1	C	USA	82,72	84,52	76	82,5	85,91	72,8
AY836524.1	A	USA	83,62	85,39	76,7	83,7	87,1	72,8
